# Multi-Inflamatuar Index is a New Predictive Parameter for Early-Term Mortality in Patients Undergoing Endovascular Aortic Repair for Ruptured Abdominal Aortic Aneurysm

**DOI:** 10.5152/eurasianjmed.2025.25736

**Published:** 2025-03-14

**Authors:** Serdar Badem, Mustafa Selçuk Atasoy, Ahmet Yüksel, Ayhan Müdüroğlu, Hakan Güven, Ali Önder Kılıç, Demir Çetintaş, Yusuf Velioğlu

**Affiliations:** 1 Department of Cardiovascular Surgery, Bursa City Hospital, Bursa, Türkiye; 2 Department of Cardiovascular Surgery, Bursa Medical Park Hospital, Bursa, Türkiye

**Keywords:** Endovascular aortic repair, multi-inflammatory index, ruptured abdominal aortic aneurysm

## Abstract

**Background:**

This study aimed to investigate the predictive value of systemic inflammation markers for mortality in patients who underwent emergency endovascular aortic repair (EVAR) due to a ruptured abdominal aortic aneurysm (rAAA).

**Methods:**

A total of 40 patients who underwent EVAR procedure due to rAAA were included in this retrospective observational cohort study. The patients were divided into 2 groups: group 1 (survivor group, n = 27) and group 2 (mortality group, n = 13). Basic clinical and demographic characteristics, hematological and biochemical parameters, and complications before the procedure were compared between the groups.

**Results:**

When the pre-procedural demographic and clinical characteristics of the groups were compared, the mean age in the mortality group and the survivor group was found to be 78.6 ± 6.2 and 67 ± 7.3 years, respectively. It was determined that the mean C-reactive protein value and median neutrophil-to-lymphocyte ratio, systemic immune-inflammatory index, inflammatory prognostic index, and multi-inflammatory index (MII) values in the mortality group were significantly higher than those in the survival group, and the mean lymphocyte, eosinophil, hemoglobin, platelet, plateletcrit, and albumin values were significantly lower than those in the survivor group. In the logistic regression analysis, only MII was found to be an independent predictor of mortality.

**Conclusion:**

Based on the results of the current study investigating a modest number of patients, high MII levels may be used as a predictor of periprocedural mortality in patients undergoing EVAR procedure due to AAAs.

**Main Points**

- Acute hemorrhagic shock and acute renal failure following the rupture of abdominal aortic aneurysm (rAAA) activate systemic inflammatory agents.
- Multi-inflammatory index significantly and independently predicts mortality after endovascular aortic repair (EVAR) due to rAAA.
- It is important for patients undergoing EVAR to be monitored frequently in the intensive care unit during the post-procedural period and to take necessary precautions early.

## Introduction

Ruptured abdominal aortic aneurysms (rAAA) have an in-hospital mortality rate of just over 50% and require urgent open repair (OR) or endovascular intervention. Treatment for rAAA is determined by the diameter, location, and symptoms of the aneurysm.^1^ Although the endovascular aortic repair (EVAR) technique is increasingly preferred for the treatment of rAAA, no significant reduction in mortality has been demonstrated compared with OR.^2-5^ Therefore, there is a need to investigate new prognostic biomarkers, identify potential measurements in higher-risk patients, and subsequent therapeutic targets to reduce mortality and morbidity rates after rAAA treatment.

In recent years, there have been many studies in the literature showing that inflammatory markers are predictors of mortality in cardiovascular diseases.^6-11^ For example, there is good evidence that hematological and biochemical systemic inflammatory markers such as C-reactive protein (CRP), neutrophil-to-lymphocyte ratio (NLR), platelet-to-lymphocyte ratio (PLR), inflammatory prognostic index (IPI), systemic immune-inflammatory index (SII), and multi-inflammatory index (MII) are both sensitive and reliable predictors of mortality in patients with unruptured AAA.^8-11^

Inflammatory prognostic index is a new prognostic biomarker created from hematological markers such as neutrophil and lymphocyte counts and biochemical markers such as CRP and albumin. (IPI = NLR × CRP/albumin) SII is a new biomarker created with NLR and platelet counts that simultaneously shows the inflammatory and immune status of patients. (SII = NLR × platelet count) MII is another newly discovered prognostic biomarker created from hematological and biochemical parameters formulated with NLR and CRP (MII = NLR × CRP). Inflammatory prognostic index, SII, and MII have been frequently used in COVID-19, oncological, and cardiovascular diseases in recent years and provide predictive information about mortality and morbidity.^12-14^ To the best of knowledge, there is no study in the literature investigating the role of IPI, SII, and MII in predicting periprocedural mortality in patients who underwent an emergency EVAR procedure due to rAAA. The aim of this study was to investigate the role of these systemic inflammatory markers in predicting perioperative mortality in patients undergoing EVAR procedure for rAAA.

## Materials and Methods

### Study Population and Design

The study was conducted in accordance with the principles of the Declaration of Helsinki, and approval was received from the Ethics Committee Board of Bursa City Hospital (Approval no: 2024-9/9; Date: 29.05.2024). All patients included in the study were given detailed information about the operation, and their written consent was obtained. In the clinic, 40 adult patients who constituted the study population underwent emergency EVAR procedure due to rAAA between May 2021 and July 2022 and were evaluated retrospectively. The patients were divided into 2 groups according to the development of in-hospital and 30-day mortality. Group 1 was composed of survivor patients (67.5%, n = 27), and group 2 was composed of patients with mortality (32.5%, n = 13). Preprocedural basic demographic and clinical characteristics of the patients, complete blood count and biochemical parameters, postprocedural complications, early and mid-term results were analyzed and compared between the groups. Exclusion criteria were as follows: patients without suitable anatomy for the EVAR procedure, patients with elective AAA, patients with poor hemodynamic status and hypovolemic shock, patients with malignancy, sepsis, autoimmune, and inflammatory diseases were excluded from the study.

### Preprocedural Evaluation

All patients underwent imaging with computed tomography angiography (CTA) with a slice thickness of 1 mm before the procedure to evaluate the abdominal aorta and visceral branches. Procedure planning and endograft sizing were performed using the RadiAnt DiCOM v2021.2 (64-bit) 3-dimensional vascular imaging tool. In this study, the indications for infrarenal rAAA intervention were accepted as the presence of symptoms, clinical examination, and CTA imaging of the rupture. In both groups of patients, proximal aortic neck length (ANL) and proximal aortic neck diameter (AND), proximal aortic neck angle (ANA), diameter of the widest region of the aneurysm, degree of calcification and thrombus in the neck region, presence of conical AN, diameters, and anatomical features of the right and left iliac arteries were evaluated. In this study, the number of patients with a neck length of less than 10 mm was 3 (23%) in the mortality group and 8 (29.6%) in the survivor group. Additionally, the number of patients with a neck diameter greater than 28 mm was 2 (15.4%) in the mortality group and 5 (18.5%) in the survivor group. Furthermore, the number of patients with a neck angle greater than 60 degrees was 4 (30.8%) in the mortality group and 11 (40.7%) in the survivor group. Three-dimensional CTA images were evaluated by at least 2 experienced cardiovascular surgeons to determine endograft dimensions. In addition, CTA images were simultaneously sent to the endograft manufacturer to confirm the measurements. Thus, the attachment points of the graft to the intact aortic region were determined. Endograft diameter and length were planned by calculating a 20% increase in graft diameter compared to the target aortic diameter.

### Procedural Technique and Post-Procedural Approach

The EVAR procedure was performed in all groups by the same surgical and anesthesia team, using the same method, after providing the necessary sterilization conditions in the angiography laboratory and under appropriate anesthesia. Before the EVAR procedure, the choice of anesthesia technique was made by taking into account the experience of the team and the characteristics of the patient. The EVAR procedure was performed using regional anesthesia and sedation in patients with poor hemodynamic status and predicted complications of general anesthesia. Both common femoral arteries were used for endograft access. Patients were administered heparin at a dose of 100 IU/kg, and when the ACT dose was over 200, the aneurysmal segment was excluded and the endograft was optimally placed on the healthy aortic wall. In all patients, stent-graft Endurant™ II (Medtronic, Santa Rosa, CA, USA) covered with polyethylene terephthalate material was used as an endograft. After the EVAR procedure, control aortography was performed to evaluate graft patency and possible endoleaks. In patients who developed endoleaks after EVAR, balloon dilatation was performed inside the endograft to support sealing. In some selected patients with appropriate anatomy, an extension stent was placed together with balloon dilatation to prevent endoleak formation. The patients were taken to the intensive care unit without any problems after the common femoral arteries were surgically closed. If the patient had any clinical complaints after the procedure, they were checked with emergency CTA. In patients without any symptoms, clinical and imaging follow-up was performed with CTA at 1, 6, and 12 months after the procedure. Patients who underwent EVAR were started on acetylsalicylic acid (ASA) along with low molecular weight heparin immediately after the procedure and throughout their hospital stay unless there were contraindications. After discharge, the patients were followed up only with ASA for antiplatelet treatment.

### Statistical Analysis

Data were entered into the Social Sciences Statistical Package (IBM® SPSS Statistics for Windows, Version 23.0, IBM SPSS Corp.; Armonk, NY, USA). The Shapiro–Wilk test was used to examine whether continuous variables showed a normal distribution. Independent Samples t-test was used to compare normally distributed continuous variables, while the Mann–Whitney *U* test was used to compare abnormally distributed continuous variables. The Chi-square test was used to compare categorical variables. Multivariate logistic regression analysis was performed to determine independent predictors of mortality. Normally distributed continuous variables were presented as mean ± standard deviation, while abnormally distributed continuous variables were presented as median (minimum–maximum). Categorical variables were presented as numbers (percentages). A *P*-value of less than .05 was considered statistically significant.

## Results

A total of 40 patients, 35 male and 5 female, were included in this study, and the mortality incidence was found to be 32.5%. It was observed that 76.9% of the patients in the mortality group and 92.6% of the patients in the survivor group were male. The mean age of the patients in the mortality group was 78.6 ± 6.2, and the mean age of the patients in the survivor group was 67 ± 7.3, and this difference was found to be statistically significant. No significant differences were found between the mortality group and the survivor group in terms of other demographic and clinical characteristics before the procedure (Table 1).

When the laboratory parameters of the patients were compared between the 2 groups, it was found that the mean CRP and median NLR, SII, MII, and IPI values were significantly higher in the mortality group than in the survivor group. In addition, it was determined that the average lymphocyte, eosinophil, hemoglobin, platelet, PCT and albumin values were significantly lower in the mortality group compared to the other group (Table 2).

When the postprocedural results of the groups were compared, general anesthesia was applied in 3 (23.1%) patients in the mortality group and in 5 (18.5%) patients in the survival group. In addition, the mean procedure time was found to be longer in the mortality group (78.7 ± 20.7 minutes) compared to the survival group (66 ± 17.6 minutes), but these differences were not statistically significant. Postprocedural Acute renal failure (ARF) developed in 8 (61.5%) patients in the mortality group and 2 (7.4%) patients in the survivor group, and this difference was found to be statistically significant (*P* < .05). In the postprocedural mortality group, 7 (53.8%) patients and in the survivor group, 6 (22.2%) patients required more blood products, and this difference was found to be statistically significant (*P* < .05). In this study, thefollow-up period was the first postprocedural month. Sixteen (40%) patients who developed intraoperative type 1a endoleaks underwent either intraprocedural balloon or extension stent graft procedures, and all patients were removed from the angiography room without any problems. No statistically significant differences were found between the groups in terms of additional procedures such as balloon and stent extension, intraprocedural complications, endoleak development, conversion to open surgery due to procedural failure, early postprocedural thromboembolectomy, ICU, and hospital stay (Table 3).

After identifying potentially important risk factors, multivariate logistic regression analysis was performed to assess the association between mortality after EVAR procedure for rAAA and independent predictors by adjusting for significant variables. According to multivariate logistic regression analysis, among advanced age, AKI, blood product use, lymphocyte, eosinophil, hemoglobin, platelet, albumin, PCT, CRP, NLR, SII, IPI, and MII, the effectiveness of advanced age and MII in predicting mortality was found to be significant (Table 4).

## Discussion

In the study, patients in the mortality group who were undergoing EVAR due to rAAA were found to be older, and postprocedural blood product use and ARF development were higher. In the univariate analysis of laboratory parameters in the mortality group, CRP, NLR, SII, MII, and IPI values were higher than in the survivor group, but the mean values of lymphocytes, eosinophils, hemoglobin, platelets, PCT, and albumin were lower. Among these parameters, only age and MII remained statistically significant in the multiple explanatory variable logistic regression analysis. The most interesting finding of this study was that MII, which is composed of hematological and biochemical parameters, significantly and independently predicted the development of mortality after EVAR procedure in rAAA patients.

Alexander et al^15^ and Healey et al^16^ stated in their studies on patients with rAAAs who were undergoing OR and EVAR procedures, that the early-term mortality rate was high in older patients. Similar to these studies, in this study, the early-term mortality rate was found to be higher in older patients who underwent EVAR procedure due to rAAA.

In the current treatment of rAAA, the results of many studies comparing EVAR with OR on postoperative mortality are still controversial.^2-5^ Duran et al^2^ compared the safety and efficacy of EVAR and OR in 37 patients with rAAA and found that the 30-day mortality rates were 8.7% and 50.0% for the EVAR and OR groups, respectively, showing that the EVAR procedure significantly reduced mortality. The IMPROVE randomized controlled trial, conducted at 30 vascular centers, compared the EVAR procedure for 613 rAAAs with OR to evaluate whether it reduces early mortality. In this large study, 30-day mortality rates were found to be 35.4% and 37.4% for the EVAR and OR groups, respectively, but they did not show statistical significance.^5^ In the AJAX study of 520 rAAA patients, 30-day mortality rates were found to be 21% in the 116 randomizable patients who were undergoing the EVAR procedure and 25% in the patients who underwent OR, and no statistically significant difference was seen in combined mortality and serious complications.^17^ Additionally, Zhang et al^18^ in their meta-analysis on 103 477 patients and 48 studies, found that the in-hospital mortality rate in rAAA patients with or without stable condition was 29.9% in the EVAR group and 40.8% in the OR group. In the second part of the same study, 383 patients from 5 articles were included, 152 patients were treated with EVAR and 231 patients with OR. They reported that the mortality of the EVAR group and the OR group was 25.7% and 46.8%, respectively.^18^ In this study, 32.5% mortality occurred in patients who underwent EVAR procedure due to rAAA. The mortality rate was affected by the use of large amounts of blood and blood products after the procedure, ARF that developed after the procedure, and the high average age of the patients. Additionally, the small number of the study population is another factor in the high mortality rate.

Multiorgan failure secondary to systemic inflammation is also an important cause of mortality in patients with hemorrhagic shock due to rAAA.^19,20^ Various hematological and biochemical parameters such as white blood cell count, NLR, PLR, and CRP have been studied in rAAA patients, and these systemic inflammatory biomarkers have been shown to predict mortality.^11^ In many studies conducted on symptomatic AAA patients treated with the OR or EVAR procedure, the prognostic value of NLR has been investigated, and it has been shown to be a very good inflammatory marker in predicting in-hospital mortality.^8-10^ Bradley et al^21^ in their review of 9 studies and 4571 patients, investigated the all-cause mortality rates of systemic inflammatory response activation in patients undergoing OR and EVAR due to AAA. According to this meta-analysis, the majority of studies identified an association between elevated NLR and increased mortality in patients undergoing both OR and EVAR. In the same study, the relationship between PLR and SII with mortality was less well defined. This study showed that activation of the systemic inflammatory response was associated with a worse prognosis in patients treated for AAA.^21^

Systemic immune-inflammatory index, IPI, and MII are derived from hematological and biochemical parameters that are noninvasive, cheap, accessible, and easily formulated and used to predict prognosis. In recent years, these biomarkers have received significant attention in cardiovascular and oncological surgeries as independent predictors of mortality and morbidity.^7,11-14^ Multi-inflammatory index, which was first reported in the literature by Casadei Gardini et al^12^, is a new inflammatory prognostic marker with high clinical value, derived from hematological and biochemical parameters NLR and CRP values to predict the prognosis in patients with metastatic colorectal cancer. To the best of knowledge, there is no published study investigating the predictive value of SII, IPI, and MII for mortality in patients undergoing EVAR due to rAAA. In this study, conducted for the first time in the literature on patients diagnosed with rAAA and undergoing the EVAR procedure, it wasfound that MII was the most significant biomarker in predicting early mortality. The study had several limitations. The study’s primary limitations were that it was retrospective and single-centered. The study’s comparatively small patient population and the limited number of parameters examined in the study were also limitations. Additionally, the lack of correlation analysis with other predictive markers of the inflammatory response was a significant limiting factor.

The study had several limitations. The primary drawbacks were its single-centered design, retrospective data collection, small sample size, and limited data analysis.

To the best of knowledge, no study has been published examining the association between IPI and mortality in patients undergoing EVAR for rAAA. The most important finding of the study was that, for the first time in the literature, MII significantly and independently predicted mortality after the EVAR procedure due to rAAA. In patients who will undergo an EVAR procedure for rAAA treatment, it is important to determine the patients at high risk of mortality by calculating the MII value before the procedure and to ensure closer follow-up and necessary precautions during and after the procedure. It is believed that the MII value should be routinely calculated and taken into account in order to develop an appropriate treatment strategy and predict complications in patients who are planned to undergo EVAR due to rAAA. However, more well-designed studies with larger patient participation rates are also needed to produce more definitive scientific evidence.

## Figures and Tables

**Table 1.** Preprocedural Baseline Demographic and Clinical Characteristics

Variable	Survivor Group (n = 27)	Mortality Group (n = 13)	*P*
Age (years)	67 ± 7.3	78.6 ± 6.2	.00*
Gender (male)	25 (92.6%)	10 (76.9%)	.16
Weight (kg)	87.4 ± 11.7	86.9 ± 10.6	.90
Height (cm)	170.5 ± 10.2	167.3 ± 5.9	.15
BMI (kg/m^2^)	30.1 ± 3.7	31.1 ± 3.8	.43
DM	3 (11.1%)	3 (23.1%)	.32
Hypertension	10 (37%)	7 (53.8%)	.31
COPD	1 (4.3%)	1 (7.7%)	.58
CAD	7 (25.9%)	4 (30.7%)	.74
CRF	2 (7.4%)	0 (0%)	.31
CHF	2 (7.4%)	1 (7.7%)	.97
PAD	3 (11.1%)	2 (15.4%)	.70
Obesity	12 (44.4%)	6 (46.1%)	.91
Smoke	11 (40.7%)	7 (53.8%)	.43
Aortic neck diameter (mm)	25.3 ± 3.5	25.8 ± 3.3	.63
Aortic neck length (mm)	13.3 ± 7.0	15.7 ± 8.6	.38
Aortic neck angle (degree)	54.9 ± 10.5	49.5 ± 10.6	.13
Widest aortic diameter (mm)	75.5 ± 13.8	82.5 ± 15	.17
Tapered neck	2 (7.4%)	2 (15.4%)	.43
Neck calcification	2 (7.4%)	1 (7.7%)	.97
LIAD (mm)	15 (9-40)	16 (11-30)	.20
RIAD (mm)	17 (9-41)	18 (11-28)	.43

BMI, body mass index; CAD, coronary artery disease; CHF, congestive heart failure; COPD, chronic obstructive pulmonary disease; CRF, chronic renal failure; DM, diabetes mellitus; HNI, hostile neck index; LIAD, left iliac artery diameter; PAD, peripheral artery disease; RIAD, right iliac artery diameter.

*Bold *P* values indicate statistical significance.

→Continues variables were presented as mean ± standard deviation or median (minimum–maximum) while categorical variables were presented as number (percentage).

**Table 2.** Laboratory Data of the Groups

Variable	Survivor Group (n = 27)	Mortality Group (n = 13)	*P*
WBC (10^3^/µL)	11.9 ± 5.2	13.9 ± 5.6	.29
Neutrophil (10^3^/µL)	8.8 ± 4.7	11.9 ± 4.9	.07
Lymphocyte (10^3^/µL)	2.2 ± 1.2	0.9 ± 0.6	.00*
Monocyte (10^3^/µL)	1 ± 0.6	0.7 ± 0.4	.15
Eosinophil (10^3^/µL)	0.14 ± 0.15	0.05 ± 0.08	.02*
Hemoglobin (g/dL)	11.3 ± 1.7	8.1 ± 1.3	.00*
RDW (fL)	43.8 ± 3.8	47.5 ± 4.8	.29
MCV (fL)	86.9 ± 5	87.9 ± 5.4	.56
MCHC (g/dL)	33.9 ± 1.1	33.3 ± 0.9	.08
PCT (%)	0.2 ± 0.08	0.1 ± 0.06	.02*
MPV (fL)	10.2 ± 0.9	10.3 ± 1.0	.69
PDW (fL)	11.3 ± 2.1	12.3 ± 2.0	.19
Platelet (10^3^/µL)	230.6 ± 95.5	168.7 ± 61.9	.02*
CRP (mg/L)	33.6 ± 33.5	152.2 ± 69.2	.00*
Glukoz (mg/dL)	197.2 ± 81	184.5 ± 74.2	.75
Creatinine (mg/dL)	1.45 (0.7-4.3)	1.47 (1.1-2.2)	.48
GFR (mL/min/1.73 m^2^)	52.9 ± 26.5	44.1 ± 10.4	.14
Albumin (g/L)	34.3 ± 5.5	27.4 ± 4.7	.00*
AST (IU/L)	18.5 (8-350)	18 (9-159)	.47
ALT (U/L)	14.5 (6-123)	13 (5-152)	.49
NLR	4.5 (0.8-14.6)	13.1 (5.2-44.7)	.00*
PLR	142.8 ± 110.8	224.5 ± 125.7	.06
SII	888.4 (247.7-3117.4)	2264.2 (329.5-5947)	.01*
MII	179.3 (30.5-758.5)	536.6 (366.8-735.5)	.00*
IPI	4.8 (1.1-22.1)	16.6 (10.7-28.2)	.00*

ALT, alanine transaminase; AST, aspartate aminotransferase; CRP, C-reactive protein; GFR, glomerular filtration rate; IPI, inflammatory prognostic index; MCHC, mean corpuscular hemoglobin concentration; MII, multi-inflammatory index; MPV, mean platelet volume; NLR, neutrophil lymphocyte ratio; PCT, plateletcrit; PDW, platelet distribution width; PLR, platelet lymphocyte ratio; RDW, red cell distribution width; SII, systemic immune-inflammatory index; WBC, white blood cell.

*Bold *P* values indicate statistical significance.

→Continues variables were presented as median (minimum–maximum).

**Table 3.** Periprocedural Data of the Groups

Variable	Survivor Group (n = 27)	Mortality Group (n = 13)	*P*
General anesthesia	5 (18.5%)	3 (23.1%)	.73
Procedure time (minutes)	66 ± 17.6	78.7 ± 20.7	.07
Additional process (balloon, extension stent, etc.)	16 (59.2%)	10 (76.9%)	.27
Intraoperative complications	0 (0%)	1 (7.7%)	.14
Conversion to open surgery	0 (0%)	0 (0%)	1.00
Thromboembolectomy	1 (4.3%)	2 (15.4%)	.18
Endograft migration	0 (0%)	0 (0%)	1.00
Endoleak	12 (44.4%)	4 (30.7%)	.40
Acute renal failure	2 (7.4%)	8 (61.5%)	.00*
Wound infection	2 (7.4%)	0 (0%)	.31
Blood product transfusion	6 (22.2%)	7 (53.8%)	.04*
ICU stay (day)	2 (1-7)	1 (1-5)	.25
Hospital stay (day)	3 (2-9)	2 (1-9)	.23

ICU, intensive care unit.

*Bold *P* values indicate statistical significance.

→Continues variables were presented as mean ± standard deviation or median (minimum–maximum) while categorical variables were presented as number (percentage).

**Table 4.** Results of Multivariate Logistic Regression Analysis to Identify Independent Predictors of Mortality Development

Variable	Odds Ratio	95% CI	*P*
Age	1.02	1.01-1.09	.02*
Acute renal failure	1.23	0.94-1.54	.66
Blood product transfusion	1.32	0.92-1.67	.82
Lymphocyte	0.75	0.66-1.11	.53
Eosinophil	0.95	0.88-1.43	.33
Hemoglobin	0.94	0.86-1.28	.14
Platelet	1.16	0.89-1.25	.88
Albumin	0.86	0.64-1.70	.27
PCT	1.17	1.08-1.69	.78
NLR	1.33	0.79-1.45	.22
CRP	1.28	0.90-1.19	.08
SII	1.40	1.08-2.25	.71
MII	1.06	1.02-1.17	.03*
IPI	1.33	1.10-1.89	.39

CRP, C-reactive protein; HNI, hostile neck index; IPI, inflammatory prognostic index; MII, multi-inflammatory index; NLR, neutrophil lymphocyte ratio; PCT, plateletcrit; RDW, red cell distribution width; SII, systemic immune-inflammatory index.

*Bold *P* values indicate statistical significance.

## Data Availability

The data that support the findings of this study are available on request from the corresponding author.
